# Breast Cancer Characteristics in Middle Eastern Women Immigrants Compared With Non-Hispanic White Women in California

**DOI:** 10.1093/jncics/pky014

**Published:** 2018-05-30

**Authors:** Clara Ziadeh, Argyrios Ziogas, Luohua Jiang, Hoda Anton-Culver

**Affiliations:** Department of Epidemiology, School of Medicine, University of California Irvine, Irvine, CA

## Abstract

**Background:**

Emerging evidence has indicated that Middle Eastern (ME) immigrants might be more likely to be diagnosed with breast cancer at advanced stage, yet have better overall survival than nonimmigrant non-Hispanic whites (NHW). This study aims to analyze the association between ME immigration status and breast cancer stage at diagnosis and survival.

**Methods:**

Using the California Cancer Registry, a total of 343 876 women diagnosed with primary in situ or invasive breast cancers were identified during 1988–2013. Multinomial logistic regression models were fitted to evaluate the risk of in situ and nonlocalized breast cancer stage in comparison with localized breast cancer among first-generation ME immigrants, second- or subsequent-generation ME immigrants, and NHW. Cox proportional hazard models were applied to calculate hazard ratios (HRs) with their 95% confidence intervals (CIs) for breast cancer mortality among the three population groups with invasive primary breast cancer.

**Results:**

First-generation ME immigrants had higher odds of being diagnosed with a nonlocalized stage (vs localized) than NHW (odds ratio [OR] = 1.17, 95% CI = 1.09 to 1.26). Second- or subsequent-generation ME immigrants also had higher odds of being diagnosed with a nonlocalized stage (vs localized) than NHW (OR = 1.31, 95% CI = 1.20 to 1.43). First-generation ME immigrants were 11% less likely to die from breast cancer than NHW (HR = 0.89, 95% CI = 0.82 to 0.97).

**Conclusions:**

First-generation ME immigrants had higher breast cancer survival despite being diagnosed at a nonlocalized breast cancer stage at diagnosis when compared with NHW. Screening interventions tailored to this ME immigrant group need to be implemented.

In the United States, breast cancer mortality has been decreasing over the past few decades. Five-year breast cancer–specific survival rates have improved from 75.2% in 1975 to 91.3% in 2009 (1). Stage at diagnosis is considered to be the strongest determinant of breast cancer survival (2). Survival rates vary by stage at diagnosis, with 100.0% for in situ, 98.5% for localized, 84.6% for regional, and 25.0% for distant breast cancers (3).

Studies have shown that immigrants to the United States present with more advanced cancer stage at diagnosis and have lower survival rates compared with nonimmigrant non-Hispanic whites (NHW) (4–9). Access to health care, lower rates of mammography screening, language barriers, genetic factors, and other sociocultural factors have been suggested to explain these disparities (10,11). Lower rates of mammography screening among immigrant women have been explained by multiple factors, including having a lower education level, being a new immigrant, and not having public insurance coverage (12). It has also been suggested that immigrants do not have a clear knowledge of the health care system, which can be a barrier in breast cancer screening (13).

One of the growing immigrant populations in the United States (14), and particularly in California (15,16), is the Middle Eastern (ME) immigrant population. Studies have been conducted to compare breast cancer stage and survival in different immigrant groups in the United States (4–10,17,18). To our knowledge, only two studies have investigated stage at diagnosis and survival in the ME immigrant population (19,20). One of the reasons is that immigrants from the Middle East are not recognized as a separate ethnic group in the US census and are combined with NHW(21). A study conducted in Michigan has shown that ME immigrants were more likely to be diagnosed at advanced stage, yet had better overall survival than NHW (19), while a study performed in California has shown similar survival patterns for stage IIA breast cancers only (20).

Cancer in different generations of immigrants has been investigated by using place of birth as an estimation for acculturation (22,23). To our knowledge, this is the first study to examine breast cancer stage at diagnosis and survival in different generations of ME immigrants in California. First-generation ME immigrants are born in the Middle East, while second- or subsequent-generation ME immigrants are born elsewhere. This study aims to analyze the association between ME immigration status and breast cancer stage at diagnosis and survival in California between 1988 and 2013.

## Methods

### Data Source

The California Cancer Registry (CCR) is California’s statewide population-based cancer surveillance system. The registry monitors incidence and death from cancer among Californians since 1988 (24). CCR captures information on the patients’ demographics, cancer characteristics, treatment, and follow-up information. The demographic information includes marital status, health insurance, and socioeconomic status (SES). The cancer characteristics include age at diagnosis, year at diagnosis, stage at diagnosis, estrogen and progesterone receptors (ER and PR), tumor grade, and cancer histology. Treatment options include surgery and chemotherapy. This study did not require institutional review board approval.

### Study Populations

This study cohort consisted of all female patients from CCR who 1) were diagnosed in California, 2) between January 1, 1988, and December 31, 2013, 3) with a primary breast cancer, 4) were younger than age 100 years at diagnosis, 5) had an available social security number (SSN), 6) were part of the three population groups of interest (first-generation ME immigrants, second- or subsequent-generation ME immigrants, and NHW), and 7) had a known breast cancer stage at diagnosis.

The three population groups of interest in this study were first-generation ME immigrants, second- or subsequent-generation ME immigrants, and NHW. If the patient had a Middle Eastern last name (25), did not have a Hispanic or Asian last name, and was born in one of the Middle Eastern countries, she was considered a first-generation ME immigrant. If the patient had a Middle Eastern last name (25), did not have a Hispanic or Asian last name, was not born in one of the Middle Eastern countries, and did not have a missing birth country, she was considered a second- or subsequent-generation ME immigrant. Finally, if the patient did not have an ME or Hispanic or Asian last name and was identified as white in the CCR data set, she was considered NHW in our analysis.

### Stage at Diagnosis and Survival

Summary stage at diagnosis existing in the CCR data set (SUMSTAGE) was used for cancer stage in this study (26). Breast cancer stage at diagnosis was categorized into in situ, localized, and nonlocalized, with nonlocalized tumors including regional and distant cancers. Regional breast cancers involve cancers that have spread to nearby lymph nodes, tissues, or organs. Distant breast cancers involve cancers that have spread to distant parts of the body. Localized cancer at diagnosis was used as the reference stage in this study.

CCR contains the patient’s underlying cause of death, vital status, and follow-up time in months. The last date for follow-up observation was December 31, 2013. Breast cancer–specific deaths were classified as codes 1740–1749 of the *International Classification of Diseases* (ICD), ninth revision, for deaths that occurred between 1988 and 1998 and codes C500–C509 of the ICD, 10th revision, for deaths that occurred in 1999 and beyond. Cancer survival analysis was completed for invasive primary breast cancer cases only; hence, in situ breast cancers were excluded from survival analysis.

### Other Study Variables

In a previous study, principal component analysis was utilized, and data from the 1990 census were used to create an SES composite score for each of the census block groups (27). These scores were sorted, categorized into quintiles, and added to the CCR data set. The lowest quintile corresponds to the lowest SES. Age at diagnosis was used as a continuous measurement, in addition to the three age categories created (<45, 45–54, and >55 years). Year at diagnosis, ranging from 1988 to 2013, was divided into five categories: 1988–1992, 1993–1997, 1998–2002, 2003–2007, and 2008–2013. ER and PR were categorized as positive, negative, and unknown. Surgery and chemotherapy treatment were categorized as no, yes, and unknown. Tumor grade was divided into five categories: well differentiated, moderately differentiated, poorly differentiated, undifferentiated/anaplastic, and unknown if differentiated. Lastly, cancer histology was categorized into ductal, lobular, ductal/lobular, mucinous, and other.

### Statistical Analysis

Descriptive data were stratified and presented for the three population groups of interest and by country of birth for first-generation ME immigrants. Means ± standard deviations and medians were presented for continuous variables and numbers (percentages) for categorical variables.

Multinomial logistic regression (28) models were fitted to evaluate the risk of in situ and nonlocalized breast cancer stage in comparison with localized cancer (reference stage) among the different generations of ME immigrants and NHW. We started with a model including age at diagnosis, year at diagnosis, and marital status (model 1). We then added SES to model 2, and health insurance to model 3.

Ten-year overall and breast cancer–specific survival percentages with 95% confidence intervals (CIs) were calculated using lifetables. The log-rank test was employed to compare survival curves among the three population groups. Cox proportional hazard models were applied to calculate hazard ratios (HRs) with their 95% confidence intervals for breast cancer–specific death among the three population groups. We began with a model including age at diagnosis, stage at diagnosis, year at diagnosis, and marital status (model 1). We then added health insurance and SES to model 2, ER and PR to model 3, and finally chemotherapy, surgery, tumor grade, and cancer histology to model 4. The proportional hazard assumption was examined by testing the interaction of time with the covariates. There was no violation for this assumption. All data analyses were completed using SAS statistical software, version 9.4 (SAS Institute Inc., Cary, NC).

## Results

**Figure 1.**
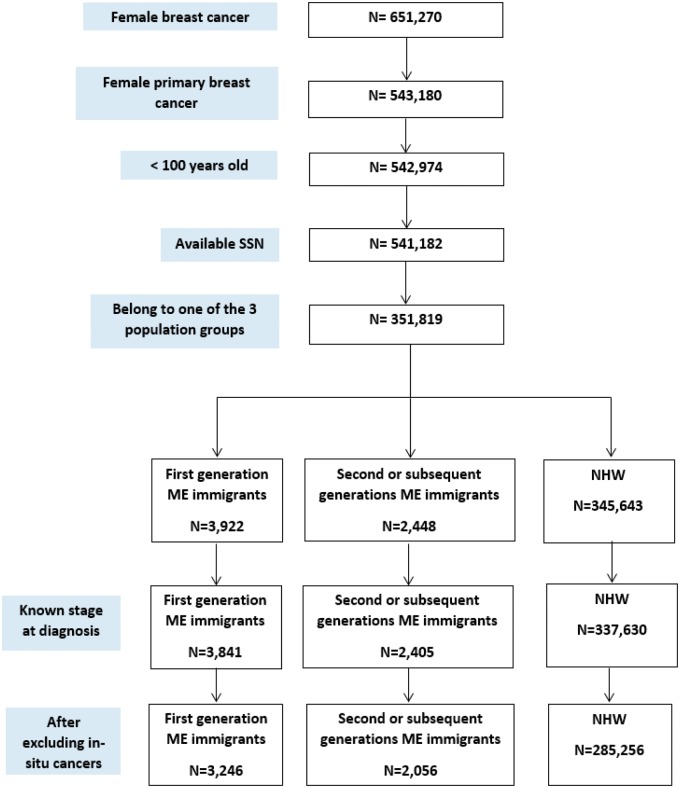
Inclusion criteria for study participants with breast cancer: California Cancer Registry, 1988–2013. CCR = California Cancer Registry; ME = Middle Eastern; NHW = non-Hispanic whites; SSN = Social Security Number.

Female breast cancer patients accounted for 651 270 of the patients in the CCR data set between 1988 and 2013, of which 543 180 female patients had primary breast cancers. We restricted eligibility to women younger than 100 years at diagnosis (n = 542 974) who had available SSNs (n = 541 182). Of those, 3922 were first-generation ME immigrants, 2448 were second- or subsequent-generation ME immigrants, and 345 643 were NHW. After excluding breast cancer cases with unknown stage at diagnosis, our sample included 3841 first-generation ME immigrants, 2405 second- or subsequent-generation ME immigrants, and 337 630 NHW women. Survival analysis was performed on invasive breast cancers only. Therefore, the final sample used in the survival analysis was 3246 breast cancer cases for first-generation ME immigrants, 2056 for second- or subsequent-generation ME immigrants, and 285 256 for NHW (Figure 1).

Table 1 shows the descriptive characteristics for breast cancer cases for all stages combined (in situ, localized, and nonlocalized), stratified by the three population groups and by country of birth for first-generation ME immigrants. The majority of first-generation ME immigrants with primary breast cancers were born in Iran, Lebanon, and Egypt. Descriptive statistics were stratified by these three countries of birth for first-generation ME immigrants. There was a difference in SES by country of birth. Of those born in Iran, 47.9% had the highest SES compared with 23.4% of those born in Egypt. The largest proportion of the cases had private health insurance (33.3%–43.8%), with the majority having positive ER (57.6%–64.7%) and PR (48.1%–54.7%). NHW were older at diagnosis and had a higher proportion of cases diagnosed at a localized stage.

**Table 1.** Descriptive characteristics of female patients with primary breast cancers, by population group and by country of birth for first-generation ME immigrants: California Cancer Registry, 1988–2013

Characteristics	First-generation ME immigrants (n = 3841)				Second- or subsequent-generation ME immigrants(n = 2405)	NHW (n = 337 630)
	Iran(n = 2150)	Lebanon(n = 336)	Egypt(n = 329)	Other ME countries*(n = 1026)		
Marital status, No. (%)						
Single	206 (9.6)	32 (9.5)	15 (4.5)	112 (10.9)	293 (12.2)	39 201 (11.6)
Married	1404 (65.3)	221 (65.8)	220 (66.9)	642 (62.6)	1509 (62.7)	191 386 (56.7)
Separated/divorced	185 (8.6)	14 (4.2)	15 (4.6)	48 (4.7)	222 (9.2)	40 213 (11.9)
Widowed	314 (14.6)	64 (19.0)	75 (22.8)	198 (19.3)	328 (13.6)	59 087 (17.5)
Unknown	41 (1.9)	5 (1.5)	4 (1.2)	26 (2.5)	53 (2.2)	7743 (2.3)
SES, No. (%)						
Lowest SES	72 (3.4)	24 (7.1)	18 (5.5)	102 (9.9)	235 (9.8)	25 916 (7.7)
Lower-middle SES	215 (10.0)	50 (14.9)	68 (20.7)	173 (16.9)	425 (17.7)	51 593 (15.3)
Middle SES	356 (16.5)	81 (24.1)	81 (24.6)	192 (18.7)	438 (18.2)	70 221 (20.8)
Higher-middle SES	477 (22.2)	80 (23.8)	85 (25.8)	264 (25.7)	551 (22.9)	85 298 (25.3)
Highest SES	1030 (47.9)	101 (30.1)	77 (23.4)	295 (28.8)	576 (31.4)	104 602 (31.0)
Health insurance, No. (%)						
Managed care, HMO, PPO, private	865 (40.2)	130 (38.7)	126 (38.3)	342 (33.3)	997 (41.5)	147 963 (43.8)
Medicaid	386 (17.9)	61 (18.1)	55 (16.7)	229 (22.3)	376 (15.6)	9753 (2.9)
Medicare	468 (21.8)	66 (19.6)	71 (21.6)	219 (21.4)	435 (18.1)	68 629 (20.3)
Insured, other type	90 (4.2)	11 (3.3)	12 (3.7)	41 (4.0)	129 (5.4)	20 368 (6.0)
Unknown if insured	299 (13.9)	60 (17.9)	59 (17.9)	185 (18.0)	433 (18.0)	89 089 (26.4)
Not insured (including self-pay)	42 (2.0)	8 (2.4)	6 (1.8)	10 (1.0)	35 (1.5)	1828 (0.5)
Age at diagnosis, y						
Mean (SD)	57.0 (12.9)	57.1 (13.1)	59.0 (12.5)	58.0 (13.7)	56.9 (13.4)	62.0 (13.7)
Median	56.0	56.0	59.0	57.5	56.0	62.0
Age at diagnosis, No. (%)						
<45 y	382 (17.8)	61 (18.1)	40 (12.2)	183 (17.9)	456 (19.0)	35 984 (10.7)
45–54 y	608 (28.3)	94 (28.0)	81 (24.6)	260 (25.3)	650 (27.0)	72 805 (21.6)
≥55 y	1160 (53.9)	181 (53.9)	208 (63.2)	583 (56.8)	1299 (54.0)	228 821 (67.8)
Stage at diagnosis, No. (%)						
In situ	363 (16.9)	57 (17.0)	36 (11.0)	139 (13.6)	349 (14.5)	52 374 (15.5)
Localized	1065 (49.5)	168 (50.0)	160 (48.6)	470 (45.8)	1122 (46.7)	184 496 (54.6)
Nonlocalized	722 (33.6)	111 (33.0)	133 (40.4)	417 (40.6)	934 (38.8)	100 760 (29.8)
Year at diagnosis, No. (%)						
1988–1992	215 (10.0)	39 (11.6)	35 (10.6)	122 (11.9)	298 (12.4)	59 662 (17.7)
1993–1997	300 (14.0)	41 (12.2)	60 (18.2)	160 (15.6)	408 (17.0)	62 727 (18.6)
1998–2002	491 (22.8)	85 (25.3)	76 (23.1)	204 (19.9)	499 (20.8)	71 795 (21.3)
2003–2007	511 (23.8)	81 (24.1)	72 (21.9)	220 (21.4)	568 (23.6)	66 572 (19.7)
2008–2013	633 (29.4)	90 (26.8)	86 (26.2)	320 (31.2)	632 (26.3)	76 874 (22.8)
ER, No. (%)						
ER positive	1390 (64.7)	213 (63.4)	206 (62.6)	653 (63.7)	1517 (63.1)	194 418 (57.6)
ER negative	267 (12.4)	44 (13.1)	50 (15.2)	153 (14.9)	337 (14.0)	43 872 (13.0)
ER unknown	493 (22.9)	79 (23.5)	73 (22.2)	220 (21.4)	551 (22.9)	99 340 (29.4)
PR, No. (%)						
PR positive	1177 (54.7)	176 (52.4)	167 (50.8)	547 (53.3)	1255 (52.2)	162 337 (48.1)
PR negative	436 (20.3)	73 (21.7)	76 (23.1)	233 (22.7)	540 (22.5)	69 122 (20.5)
PR unknown	537 (24.0)	87 (25.9)	86 (26.1)	246 (24.0)	610 (25.4)	106 171 (31.5)
Tumor grade, No. (%)						
Well differentiated	319 (14.8)	48 (14.3)	47 (14.3)	130 (12.7)	318 (13.2)	58 650 (17.4)
Moderately well differentiated	807 (37.5)	120 (35.7)	125 (38.0)	385 (37.5)	829 (34.5)	117 972 (34.9)
Poorly differentiated	649 (30.2)	101 (30.1)	102 (31.0)	328 (32.0)	798 (33.2)	83 986 (24.9)
Undifferentiated/anaplastic	79 (3.7)	17 (5.1)	5 (1.5)	37 (3.6)	103 (4.3)	11 231 (3.3)
Unknown if differentiated	296 (13.7)	50 (14.9)	50 (15.2)	146 (14.2)	357 (14.8)	65 791 (19.5)
Histologic type, No. (%)						
Ductal	1324 (61.6)	222 (66.1)	204 (62.0)	665 (64.8)	1545 (64.2)	220 543 (65.3)
Lobular	178 (8.3)	22 (6.6)	27 (8.2)	81 (7.9)	178 (7.4)	30 153 (8.9)
Ductal/lobular	234 (10.9)	29 (8.6)	25 (7.6)	82 (8.0)	208 (8.7)	23 796 (7.1)
Mucinous	27 (1.3)	3 (0.9)	12 (3.7)	26 (2.5)	39 (1.6)	6492 (1.9)
Others	387 (18.0)	60 (17.9)	61 (18.5)	172 (16.8)	435 (18.1)	56 646 (16.8)
Surgery, No. (%)						
No	60 (2.8)	19 (5.6)	25 (7.6)	49 (4.8)	122 (5.1)	14 032 (4.2)
Yes	2090 (97.2)	317 (94.4)	304 (92.4)	976 (95.1)	2283 (94.9)	323 508 (95.8)
Unknown	0 (0.0)	0 (0.0)	0 (0.0)	1 (0.1)	0 (0.0)	90 (0.0)
Chemotherapy, No. (%)						
No	1357 (63.1)	202 (60.0)	196 (59.6)	621 (60.5)	1429 (59.4)	239 355 (70.9)
Yes	757 (35.2)	125 (37.2)	128 (38.9)	389 (37.9)	943 (39.2)	93 176 (27.6)
Unknown	36 (1.7)	9 (2.7)	5 (1.5)	16 (1.6)	33 (1.4)	5099 (1.5)

* Other ME countries = Afghanistan, Armenia, Algeria, Iraq, Israel, Jordan, Morocco, Pakistan, Saudi Arabia, Sudan, Somalia, Syria, Tunisia, Turkey, and Yemen. ER = estrogen receptor; ME = Middle Eastern; NHW = non-Hispanic white; PR = progesterone receptor; SES = socioeconomic status.

Results from the multinomial logistic regression are illustrated in Table 2. First-generation ME immigrants had higher odds of being diagnosed with a nonlocalized stage (vs localized stage) when compared with NHW (odds ratio [OR] = 1.17, 95% CI = 1.09 to 1.26), after adjusting for age at diagnosis, year at diagnosis, marital status, SES, and health insurance. Second- or subsequent-generation ME immigrants also had higher odds of being diagnosed with a nonlocalized stage (vs localized stage) when compared with NHW (OR = 1.31, 95% CI = 1.20 to 1.43). No statistically significant differences were detected in the odds of being diagnosed with in situ breast cancers (vs localized stage) between ME immigrants and NHW or in the odds of being diagnosed with nonlocalized stage (vs localized stage) among the different generations of ME immigrants.

**Table 2.** ORs and 95% CIs in first-generation ME immigrants compared with NHW and second- or subsequent-generation ME immigrants, and in second- or subsequent-generation ME immigrants compared with NHW, for in situ and nonlocalized breast cancer stages compared with localized stages: California Cancer Registry, 1988–2013*

	In situ vs localized	Nonlocalized vs localized
Statistical models and variables	OR (95% CI)	OR (95% CI)
First-generation ME immigrants compared with NHW		
Model 1: age at diagnosis, year at diagnosis, and marital status	0.98 (0.89 to 1.07)	1.28 (1.20 to 1.38)
Model 2: model 1 + SES	0.97 (0.88 to 1.07)	1.30 (1.21 to 1.39)
Model 3: model 2 + health insurance	1.02 (0.93 to 1.12)	1.17 (1.09 to 1.26)
Second- or subsequent-generation ME immigrants compared with NHW		
Model 1: age at diagnosis, year at diagnosis, and marital status	0.97 (0.86 to 1.09)	1.42 (1.30 to 1.55)
Model 2: model 1 + SES	0.97 (0.86 to 1.10)	1.41 (1.29 to 1.54)
Model 3: model 2 + health insurance	1.01 (0.89 to 1.14)	1.31 (1.20 to 1.43)
First-generation compared with second- or subsequent-generation ME immigrants		
Model 1: age at diagnosis, year at diagnosis, and marital status	1.01 (0.87 to 1.18)	0.90 (0.80 to 1.01)
Model 2: model 1 + SES	1.00 (0.86 to 1.17)	0.92 (0.82 to 1.03)
Model 3: model 2 + health insurance	1.03 (0.88 to 1.20)	0.89 (0.80 to 1.00)

* Model 1: adjusted by age at diagnosis, year at diagnosis, and marital status. Model 2: adjusted by age at diagnosis, year at diagnosis, marital status, and SES. Model 3: adjusted by age at diagnosis, year at diagnosis, marital status, SES, and health insurance. Localized stage is the baseline or referent stage. CI = confidence interval; ME = Middle Eastern; NHW = non-Hispanic white; OR = odds ratio; PR = progesterone receptor; SES = socioeconomic status.

The 10-year overall and breast cancer–specific survival analyses are illustrated in Table 3. Regardless of the breast cancer diagnosis stage, first-generation ME immigrants had the highest overall survival, while NHW had the lowest overall survival among the three population groups. First-generation ME immigrants also had the highest breast cancer–specific survival among the three population groups for localized and nonlocalized breast cancer stages. Survival percentages from breast cancer were higher than overall survival. Nonlocalized breast cancer cases had lower survival when compared with localized breast cancers. The log-rank test was computed, and it showed a statistically significant difference among the three population groups, except for breast cancer–specific survival in localized cancer stage.

**Table 3.** Ten-year overall and breast cancer–specific survival for primary female invasive breast cancers for stages combined and stratified by breast cancer stage in the three population groups: California Cancer Registry, 1988–2013*

Stratification categories	No. of patients	10-y overall survival		10-y breast cancer–specific survival	
		No. of deaths	Survival % (95% CI)	No. of deaths from breast cancer	Survival % (95% CI)
Stages combined					
First-generation ME immigrants	3246	937	60.0 (57.9 to 62.1)	531	77.4 (75.6 to 79.2)
Second- or subsequent-generation ME immigrants	2056	746	51.7 (49.1 to 54.4)	417	72.7 (70.3 to 75.1)
NHW	285 256	123 617	45.0 (44.7 to 45.2)	46 898	78.2 (78.0 to 78.4)
Log-rank test *P*		<.0001		<.0001	
Localized stage					
First-generation ME immigrants	1863	354	71.0 (68.4 to 73.6)	119	90.4 (88.6 to 92.1)
Second- or subsequent-generation ME immigrants	1122	293	62.3 (58.8 to 65.9)	89	87.9 (85.5 to 90.4)
NHW	184 496	69 779	50.0 (49.7 to 50.2)	14 200	88.9 (88.7 to 89.1)
Log-rank test *P*		<.0001		.1808	
Nonlocalized stage					
First-generation ME immigrants	1383	583	45.5 (42.3 to 48.7)	412	59.9 (56.7 to 63.1)
Second- or subsequent-generation ME immigrants	934	453	39.0 (35.2 to 42.8)	328	54.2 (50.3 to 58.2)
NHW	100 760	53 838	35.8 (35.5 to 36.2)	32 698	58.6 (58.2 to 58.9)
Log-rank test *P*		<.0001		.0234	

* CI = confidence interval; ME = Middle Eastern; NHW = non-Hispanic white.

After adjusting for age at diagnosis, stage at diagnosis, year at diagnosis, marital status, health insurance, SES, ER, PR, chemotherapy, surgery, tumor grade, and cancer histology, first-generation ME immigrants were 11% less likely to die from breast cancer than NHW (HR = 0.89 with 95% CI = 0.82 to 0.97). There were no statistical differences in breast cancer death rates between second- or subsequent-generation ME immigrants and NHW (HR = 1.03, 95% CI = 0.93 to 1.13). First-generation ME immigrants were less likely to die from breast cancer than second- or subsequent-generation immigrants. However, in the final model after full adjustment, the difference was marginally statistically significant (HR = 0.88, 95% CI = 0.77 to 1.00) (Table 4).

**Table 4.** HRs and 95% CIs for breast cancer–specific mortality in first-generation ME immigrants compared with NHW and second- or subsequent-generation ME immigrants, and in second- or subsequent-generation ME immigrants compared with NHW: California Cancer Registry, 1988–2013*

	First-generation ME immigrants compared with NHW (n = 288 502)	Second- or subsequent-generation ME immigrants compared with NHW (n = 287 312)	First-generation compared with second- or subsequent-generation ME immigrants (n = 5302)
Statistical models and variables	HR (95% CI)	HR (95% CI)	HR (95% CI)
Model 1: age at diagnosis, stage at diagnosis, year at diagnosis, and marital status	0.98 (0.90 to 1.07)	1.21 (1.10 to 1.33)	0.83 (0.73 to 0.94)
Model 2: model 1 + health insurance and SES	0.85 (0.78 to 0.93)	1.05 (0.95 to 1.16)	0.84 (0.73 to 0.95)
Model 3: model 2 + ER and PR	0.86 (0.79 to 0.94)	1.07 (0.97 to 1.18)	0.83 (0.73 to 0.94)
Model 4: model 3 + chemotherapy treatment, surgery, tumor grade, and cancer histology	0.89 (0.82 to 0.97)	1.03 (0.93 to 1.13)	0.88 (0.77 to 1.00)

* Model 1: adjusted by age at diagnosis, stage at diagnosis, year at diagnosis, and marital status. Model 2: adjusted by age at diagnosis, stage at diagnosis, year at diagnosis, marital status, health insurance, and SES. Model 3: adjusted by age at diagnosis, stage at diagnosis, year at diagnosis, marital status, health insurance, SES, ER, and PR. Model 4: adjusted by age at diagnosis, stage at diagnosis, year at diagnosis, marital status, health insurance, SES, ER, PR, chemotherapy, surgery, tumor grade, and cancer histology. CI = confidence interval; ER = estrogen receptor; HR = hazard ratio; ME = Middle Eastern; NHW = non-Hispanic white; PR = progesterone receptor; SES = socioeconomic status.

## Discussion

This study found that first-generation ME immigrants had higher breast cancer survival despite being diagnosed at a nonlocalized breast cancer stage when compared with NHW.

Previous studies have shown that immigrants present with more advanced breast cancer stage at diagnosis (4,6,7,29–31). Our results are similar, with first-generation ME immigrants having higher odds of nonlocalized breast cancer stage when compared with NHW. A comparative survey among four ME registries and the United States showed more than 45% of ME registry participants (except Israel-Jewish area) being diagnosed with breast cancer at a regional stage (32). Multiple factors have been reported to contribute to this advanced stage at diagnosis in immigrants. These factors included lower mammography screening rates (33,34), lower SES, different cultural beliefs (35), and limited access to health care (36). Studies have been conducted to look at predictors of mammography screening and breast cancer examination in immigrant groups. These predictors included having health insurance, having higher income, longer duration of residency in the United States, and greater acculturation (37). Reasons for mammogram noncompliance included not having previous mammograms, fear of mammography, and lack of time to take the test (38). A report from Jordan showed that only 7% of the 1549 population-based randomly selected women, who were 18 years and older, ever had a mammogram (39). Studies have been conducted to understand factors influencing breast cancer screening and examination in ME women. These factors included perceived importance of mammography, intent to be screened, and religious/cultural restrictions (40–46). We hypothesized that a potential reason for first-generation ME immigrants to be diagnosed with advanced breast cancer stage at diagnosis might be the lack of access to health care. However, our results showed that even after adjusting for SES and health insurance, first-generation ME immigrants still had higher odds of being diagnosed with nonlocalized stage compared with NHW. Cultural and immigration-related barriers might be responsible for these findings, as shown in a study conducted in the Washington, DC, area on Jordanian and Palestinian first-generation immigrants (47). ME women tend to get very busy in their houses, prioritize their families, and not go to the clinician until symptoms appear. Women from the Middle East have their own beliefs in Allah’s Will. In some cases, they get strong objections from their partners and their families on getting seen by a clinician (particularly a male clinician). Exposing their female body is forbidden by their Islamic religion. Lastly, they do not have a habit of getting annual check-ups, are not motivated in screening, and have a deep fear of cancer (47).

This study also showed first-generation ME immigrants having higher breast cancer survival when compared with NHW. Our results are similar to the limited literature conducted on ME immigrants in the United States (19,20). This higher survival in first-generation ME immigrants may be explained by their social support and adherence to a Mediterranean diet. Studies have shown that women with an increase in their social support system after breast cancer diagnosis have higher survival rates (48). Furthermore, the absence of emotional support increases the risk of dying from breast cancer (49). Family is the fundamental social unit in ME families (50–52). After cancer diagnosis, ME culture play a role as the patients’ caregivers. ME families often provide emotional and social support. This can help increase the chance of survival from breast cancer for first-generation ME patients. The higher survival in first-generation ME immigrants can also be explained by their adaptation of the Mediterranean diet. Studies have shown that adherence to a Mediterranean diet is associated with higher survival (53,54). The lower mortality pattern in immigrants has also been studied in the Latino community, where two different hypotheses have been suggested and tested: salmon bias and healthy migrant effect (55). Salmon bias, where immigrants tend to return back home to die when they are diagnosed with terminal cancer, has been considered as an explanation for lower mortality in different immigrant groups including ME immigrants traveling back to Europe (56). The United States is geographically close to Mexico, and so is Europe to the ME countries. We speculate that the lower mortality in ME first-generation immigrants is not due to salmon bias given the long travel distance between the United States and the countries of the Middle East. However, this lower mortality can be explained by the healthy migrant effect, where healthier ME people immigrate to the United States.

In this study, we assessed whether acculturation is associated with breast cancer stage at diagnosis and survival by investigating place of birth and looking at different generations of ME immigrants (23). Second- or subsequent-generation ME immigrants had higher odds of being diagnosed with nonlocalized breast cancer stage when compared with NHW. We believe that the same cultural barriers preventing first-generation ME immigrants from being screened are possible explanations for the observed advanced cancer stage in second- or subsequent-generation ME immigrants. This was further demonstrated by the absence of stage differences between the different generations of ME immigrants. However, no statistically significant differences exist in breast cancer mortality between second- or subsequent-generation ME immigrants and NHW, suggesting the impact of acculturation on breast cancer survival. Second- or subsequent-generation ME immigrants tend to adopt a Westernized diet, which is positively associated with higher mortality (57). This was also shown by first-generation ME immigrants having a survival advantage (although marginal) over second- or subsequent-generation ME immigrants.

This is the first study to investigate breast cancer stage and survival in different generations of ME immigrants in California. We used CCR, California’s statewide cancer registry, which captures cancer incidence and characteristics among Californians since 1988. Our study also bares a few limitations. Women who had an ME maiden name but changed their last name after marriage or children born to ME women but not ME men were not captured in this study. In addition, we were not able to identify ME immigrants with missing ME last names or patients with missing places of birth. Data on human epidermal growth factor receptor 2 was missing in more than 60% of the cases; therefore, we did not include this variable in the analysis. Our study lacks information on reproductive factors (nulliparity, early menarche, and late menopause), which are known to increase breast cancer risk. It also lacks information on body mass index, smoking, alcohol consumption, and diet. Immigrants tend to adopt a Westernized diet after immigration or with further generations. Data on other comorbid conditions were not available in CCR. These comorbidities could have clarified some of the survival patterns seen in this study. We could not measure time since immigration for first-generation ME immigrants. Lastly, there was a statistically significant difference in sample sizes among the three population groups, limiting the comparability of our groups.

In summary, first-generation ME immigrants were diagnosed at a nonlocalized breast cancer stage at diagnosis when compared with NHW. However, they had higher breast cancer survival. Other studies are needed to confirm our results. Furthermore, screening interventions conducted in an appropriate language and tailored to this ME immigrant group, taking into consideration their specific cultural beliefs, need to be implemented. Considerations should be made to start breast cancer screening at a younger age in ME immigrants (58,59), and perhaps to screen more frequently.

## Funding

No funding sources reported.

## Note

Affiliation of authors: Department of Epidemiology, School of Medicine, University of California Irvine, Irvine, CA.
